# The effect of uncertainty in patient classification on diagnostic performance estimations

**DOI:** 10.1371/journal.pone.0217146

**Published:** 2019-05-22

**Authors:** Leo C. McHugh, Kevin Snyder, Thomas D. Yager

**Affiliations:** 1 Immunexpress, Inc., Seattle, Washington, United States of America; 2 Center for Drug Evaluation and Research, United States Food and Drug Administration, Silver Spring, Maryland, United States of America; University of Padova, ITALY

## Abstract

**Background:**

The performance of a new diagnostic test is typically evaluated against a comparator which is assumed to correspond closely to some true state of interest. Judgments about the new test’s performance are based on the differences between the outputs of the test and comparator. It is commonly assumed that a small amount of uncertainty in the comparator’s classifications will negligibly affect the measured performance of a diagnostic test.

**Methods:**

Simulated datasets were generated to represent typical diagnostic scenarios. Comparator noise was introduced in the form of random misclassifications, and the effect on the apparent performance of the diagnostic test was determined. An actual dataset from a clinical trial on a new diagnostic test for sepsis was also analyzed.

**Results:**

We demonstrate that as little as 5% misclassification of patients by the comparator can be enough to statistically invalidate performance estimates such as sensitivity, specificity and area under the receiver operating characteristic curve, if this uncertainty is not measured and taken into account. This distortion effect is found to increase non-linearly with comparator uncertainty, under some common diagnostic scenarios. For clinical populations exhibiting high degrees of classification uncertainty, failure to measure and account for this effect will introduce significant risks of drawing false conclusions. The effect of classification uncertainty is magnified further for high performing tests that would otherwise reach near-perfection in diagnostic evaluation trials. A requirement of very high diagnostic performance for clinical adoption, such as a 99% sensitivity, can be rendered nearly unachievable even for a perfect test, if the comparator diagnosis contains even small amounts of uncertainty. This paper and an accompanying online simulation tool demonstrate the effect of classification uncertainty on the apparent performance of tests across a range of typical diagnostic scenarios. Both simulated and real datasets are used to show the degradation of apparent test performance as comparator uncertainty increases.

**Conclusions:**

Overall, a 5% or greater misclassification rate by the comparator can lead to significant underestimation of true test performance. An online simulation tool allows researchers to explore this effect using their own trial parameters (https://imperfect-gold-standard.shinyapps.io/classification-noise/) and the source code is freely available (https://github.com/ksny/Imperfect-Gold-Standard).

## Introduction

The performance of a new diagnostic test is typically evaluated against a comparator or ‘gold standard’ which is assumed to correspond closely to some true state of interest (‘Ground Truth’). Judgments about the performance of the new test are based on the differences between the outputs of the test and its comparator. In many contexts, however, the comparator itself may be imperfect. Examples of imperfect comparators are common in medical diagnostics. These include comparators for which the measured value is precise but imperfectly represents the state of the underlying condition, such as serum creatinine for diagnosing kidney injury [1]; comparators for which the measurements are variable but interpretation of the result is not variable such as the diagnosis of hypertension [2]; and comparators for which accurate measurements are taken that truly represent the true state of the patient, but for which inconsistency exists in the interpretation of results, such as the diagnosis of pneumonia by chest X-ray [3]. These sources of variability in the comparator introduce a problem in the interpretation of data generated by a new diagnostic test. It may not be possible to know whether discrepancies between the results produced by the test and the comparator are due to inaccuracy of the test, inaccuracy of the comparator, or both. In such cases the measured performance of the new test relative to that of the comparator will not be an accurate indicator of the true test performance.

It is commonly assumed that a small amount of uncertainty in classification by the comparator will be of negligible consequence, when measuring the performance of a diagnostic test. The present work critically examines this assumption in a variety of contexts, and shows it to be generally false, especially for tests that are required to have very high performance. Non-statistician medical experts may underappreciate the magnitude of the effect of even small amounts of comparator uncertainty on apparent test performance. Consequently, in diagnostic evaluation studies, comparator uncertainty may not always be identified or accounted for in the analysis or interpretation of results, thus risking erroneous or biased conclusions (see, for example, reference [4] and references 18–25 therein). The purpose of the present study is not to develop theory to allow calculation of this effect, as theory is already well researched and established [5,6]. Rather we seek to further develop two practical aspects: 1) to explore, by way of specific examples, the consequence and magnitude of the effect of comparator uncertainty on the apparent performance of binary tests, in various diagnostic settings; and 2) to present a simulation tool that will be useful for clinical trial stakeholders who might not have the specialized statistical training or tools needed to estimate the effect of comparator classification uncertainty, yet who nonetheless need to understand this effect for the correct interpretation of trials. The accompanying simulation tool (https://imperfect-gold-standard.shinyapps.io/classification-noise/) will allow the non-statistician medical expert to easily conduct simulations to further explore the effects of comparator uncertainty, without the need for advanced statistical training. Some of the results of our study have been previously reported in the form of an abstract [7].

## Definitions

*Call*: A positive or negative classification or designation, derived or provided by any method, algorithm, test or device. For example, a test result falling above a given threshold could be considered a positive call, and a clinician’s opinion that a patient is disease-free could be considered a negative call.

*Comparator*: a previously established test method, against which the results from a new test will be compared. ‘Comparator’ is used in preference to ‘gold standard’ or ‘reference method’ to signify that the results from the comparator may diverge significantly from the Ground Truth.

*Ground Truth*: the true positive or negative state of a subject, in a binary classification scheme.

*Negative Percent Agreement (NPA)*: The percentage of comparator negative calls that are called as negative by the test under evaluation. This value is calculated identically to specificity. However, NPA is used in place of specificity to recognize the fact that due to the uncertain comparator, this measure should not be construed to accurately reflect the measurement that specificity presumes.

*Positive Percent Agreement (PPA)*: The percentage of comparator positive calls that are called as positive by the test under evaluation. This value is calculated identically to sensitivity. However, PPA is used in place of sensitivity to recognize the fact that due to the uncertain comparator, this measure should not be construed to accurately reflect the measurement that sensitivity presumes.

## Theory

The effect of classification uncertainty on apparent test performance is known variously as ‘information bias’, ‘misclassification bias’ or ‘non-differential bias’ in medicine and epidemiology and goes by other names in other fields [8–10]. These terms refer to the fact that as classification uncertainty increases, an increasingly large gap will appear between the true performance of the test and empirical measures of test performance such as sensitivity, specificity, negative predictive value (NPV), positive predictive value (PPV), or area under the receiver operating characteristic curve (ROC AUC). It has been recognized for many years that the imperfection of available comparators constitutes a source of difficulty in the evaluation of new diagnostic tests [11–16]. The more recent literature describes a number of examples in which the use of imperfect comparators has led to complications in evaluating the performance of new diagnostic tests for conditions as varied as carpal tunnel syndrome [17], kidney injury [1,18] and leptospirosis [19].

### Reference bias and classification noise

In general, discrepancies between a comparator and the true state it purports to measure may arise from two sources: reference bias and classification noise.

*Reference bias* is the tendency of a comparator to produce values that fall systematically to one side of the true state being evaluated, resulting in consistent misclassification of patients on the basis of known or unknown patient characteristics. For example, multiple clinicians may agree on a diagnosis based upon reference to a single comparator, but if the comparator is biased or deficient in some way, then we would expect the agreed-upon cases to have a common tendency to be incorrect. Similarly, if one of the variables used to place subjects into categories is overly weighted, then the comparator will be biased away from the true state and towards the over-weighted variable. As a third example, reference bias may also occur when there is incomplete representation (i.e. missing data) for some of the variables used for classification. This will lead to reference bias away from the incompletely specified variables, and towards those variables that have greatest representation in the dataset. A fourth example of reference bias may occur when multiple clinicians are consulted for a medical diagnosis. If, for a patient, each clinician can see the diagnoses made by the previous clinicians, this could lead to a reference bias in the direction of the earlier diagnoses. A fifth example of reference bias is automation bias, where a software algorithm consistently makes the same mistakes in the same kinds of patients, such as in the automated diagnosis of electrocardiograms [20]. Reference bias is particularly difficult to detect because multiple independent comparators (for example independent clinicians’ diagnoses) may be consistent with each other, giving the appearance of being correct, yet may be incorrect nonetheless. Furthermore, because the Ground Truth cannot be known directly, estimating the magnitude of reference bias is difficult. Approaches such as discrepant analysis [21] have been proposed, for estimating the magnitude of reference bias in particular situations.

It is also worth noting that in rare cases, reference bias can lead to inflation of the apparent performance of a test, as described in S1 Supporting Information (“Example of reference bias”). This can occur when the same manner of classification bias is present in both the comparator and the new diagnostic test under evaluation, leading to correlated misclassifications of the same patients. The risk of reference bias-induced performance inflation is only relevant under the conditions of low noise in both the new test and the comparator. This is because noise will decouple the agreements produced by the correlated biases in the new test and comparator. In this paper we acknowledge but do not further explore reference bias because is it not usually possible to measure it. However, reference bias can be seen as an additional potential contributor to under-estimation of diagnostic performance in datasets such as those considered in this paper.

*Classification noise* is the other fundamental cause of differences between a comparator and the Ground Truth it is supposed to represent. It can be viewed as the amount of uncertainty inherent in the classifications produced by the comparator. Classification noise can be intuitively understood by considering that if a comparator diagnosis is uncertain, and if a number of similar cases are presented and similarly classified, we would expect some of them to be wrong (but would not know which ones). We define classification noise as instability (random variation) in the comparator, which produces randomly scattered values on either side of the true state. Classification noise is not attributed to systematic causes, but rather to stochastic processes or to the absence of information that would close the gap between measured values and Ground Truth.

Noise in a comparator can be estimated by applying the comparator multiple times to the same patient or sample, or to replicate samples, and then observing the variation between results. (The replicate results would be identical if the comparator did not contain noise.) For example, in a situation where a comparator consists of the consensus of expert opinions, different clinicians making diagnoses may interpret the same information differently, leading to different diagnoses for the same patient. In defining a comparator, attempts should be made to minimize the amount of classification noise. However, because the comparator is based ultimately on experimental measurements or empirical assessments, it will not be possible to remove all classification noise.

### Uncertainty and misclassification events

Uncertainty in patient classification can be measured in a number of ways, most commonly by an inter-observer agreement statistic such as Cohen’s Kappa, or by the correlation terms in a Multitrait-Multimethod Matrix. These and related statistics estimate the extent of agreement in classifying the same patients or samples by different tests or reviewers, relative to the extent of agreement that would be expected at random. Cohen’s Kappa ranges from 0 to 1. A value of 1 indicates perfect agreement, and values of less than 0.65 are generally interpreted to mean that there is a high degree of variability in classifying the same patients or samples. Kappa values are often used to describe inter-rater reliability (i.e. the same patient between clinicians) and intra-rater reliability (i.e. the same patient with the same clinician on different days). Kappa values can also be used to estimate the variability in test measurements, such as between commercially available at-home pregnancy tests. Variability in patient classification can also be captured directly as a probability, as in standard Bayesian analysis. Irrespective of which metric is used to capture the variability in classification, there is a direct correspondence between the measured variability in a test or comparator, the uncertainty reflected in that measurement, and the misclassifications that occur as a result of this uncertainty.

Generally speaking, a known amount of uncertainty will correspond to an exact expected misclassification rate. However, for diagnosis in any specific patient cohort, an observer will not know for sure which patients have been misdiagnosed, or even how many have been misclassified. For example, if a test is used for binary classification and we know that a negative call for a test is 95% accurate, then for each patient classified as negative there will be a 5% chance that the patient is actually positive. The random nature of the uncertainty means that for 100 patients in a trial who have been called negative by the test, we expect 5% to be misclassified, but it could be that actually in the trial 10 are misclassified, or that none are (although both of these alternatives are relatively unlikely). The misclassification rate for a trial can be estimated in a number of ways, such as repeat testing of samples, comparison to another test of presumed greater accuracy, or inferring an expected error rate from other sources of information.

### Example: Smeared distribution of output values

**Fig 1:**
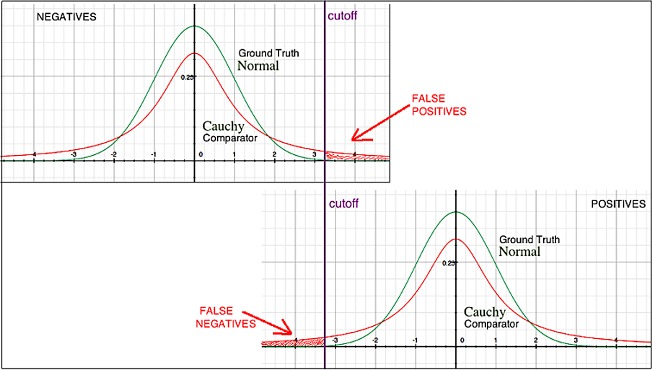
Example illustrating the problem of noise in a comparator.

A comparator might have an inherent property or limitation that causes it to return a broadened distribution of values, as compared to the Ground Truth that is being measured. An example is shown in Fig 1. Suppose that a particular condition is characterized by a variable having a continuous normal distribution at the level of Ground Truth, and that cutoffs have been defined to identify rare events (positive or negative calls) at the tails of the distribution. Suppose also that a comparator used to repeatedly measure this condition returns a Cauchy distribution of values. Then the distribution of measured values will have extended tails, not present at the Ground Truth level, which could lead to either false positive or false negative calls being made by the comparator. See Fig 1 and also references [22,23].

With increasing noise in a comparator, the totality of observed differences between a diagnostic test under evaluation and the comparator will increase. The consequence is that any new diagnostic test will appear to perform better relative to a comparator containing less noise, and worse relative to a comparator containing more noise. Consequently, the new test may appear to exhibit different levels of performance in different populations or settings where the amount of comparator noise can vary [24].

## Methods

### Simulated data

Simulated datasets were generated to represent typical diagnostic scenarios, and are presented as a ‘starting point’ assuming no classification error. Comparator noise was then introduced in the form of random misclassifications. The effect on the apparent performance of the diagnostic test was determined, as the amount of comparator noise was increased. To present a statistically valid representation of the randomness of noise injection, each amount of noise was randomly introduced in 100 iterations and the aggregate results are shown. The same simulation methodology used to generate the figures discussed below has also been implemented in an online simulation tool allows researchers to explore the comparator noise effect using their own trial parameters: https://imperfect-gold-standard.shinyapps.io/classification-noise/. The source code for the simulation tool has been made publicly available: https://github.com/ksny/Imperfect-Gold-Standard.

### Actual data

We also consider data from a study conducted in the USA and Netherlands on a new sepsis diagnostic test [25]. Three independent diagnoses per patient were rendered by expert panelists on the basis of information contained in case report forms, and the combination of diagnoses was used to determine the overall confidence of classification for each patient as detailed in S2 Supporting Information (“Method to estimate the confidence of patient classifications by an expert panel comparator”). Misclassifications were introduced randomly, weighted by the uncertainty distribution observed in patient classification as described in S3 Supporting Information (“Weighting for introduced misclassification events”). To present a statistically valid representation of the randomness of selection, each injection of classification noise was randomly drawn from the uncertainty distribution observed in the trial and introduced in 100 iterations, and the aggregate results are shown. Four different selections of patients from the total trial enrollment (N = 447) were made, and were analyzed separately: (1) the subset of patients (N = 290; 64.9% of total) who received unanimous concordant diagnoses by the external expert panelists, and who were also assigned the same diagnosis by the study investigators at the clinical sites where the patients originated. We deemed this the “super-unanimous” group, and assumed that when the external expert panelists and the study investigators at the clinical sites agreed, the diagnoses were more likely to be correct. These patients represent the stratum of the trial cohort with the lowest probability of error in the comparator; (2) the subset of patients (N = 410; 91.7% of total) who received a consensus (majority) diagnosis by the external panel. This patient subset excluded 37 patients who were classified as ‘indeterminate’ because a consensus diagnosis could not be reached by the expert panelists; (3) the set of all patients (N = 447) with a forced diagnosis of either positive or negative, regardless of the degree of uncertainty associated with each patient; (4) the subset of patients with clinical notes indicating respiratory related illness (N = 93; 20.8% of total) for whom a relatively high level of classification uncertainty was expected and observed. Each of these four selections of patients had an expected misclassification rate determined by the mean of the residual uncertainty averaged over the three external panelists’s assessments, as detailed in S4 Supporting Information (“Calculating misclassification rates, based on patient confidence values”).

## Results

### Simulated data

**Fig 2:**
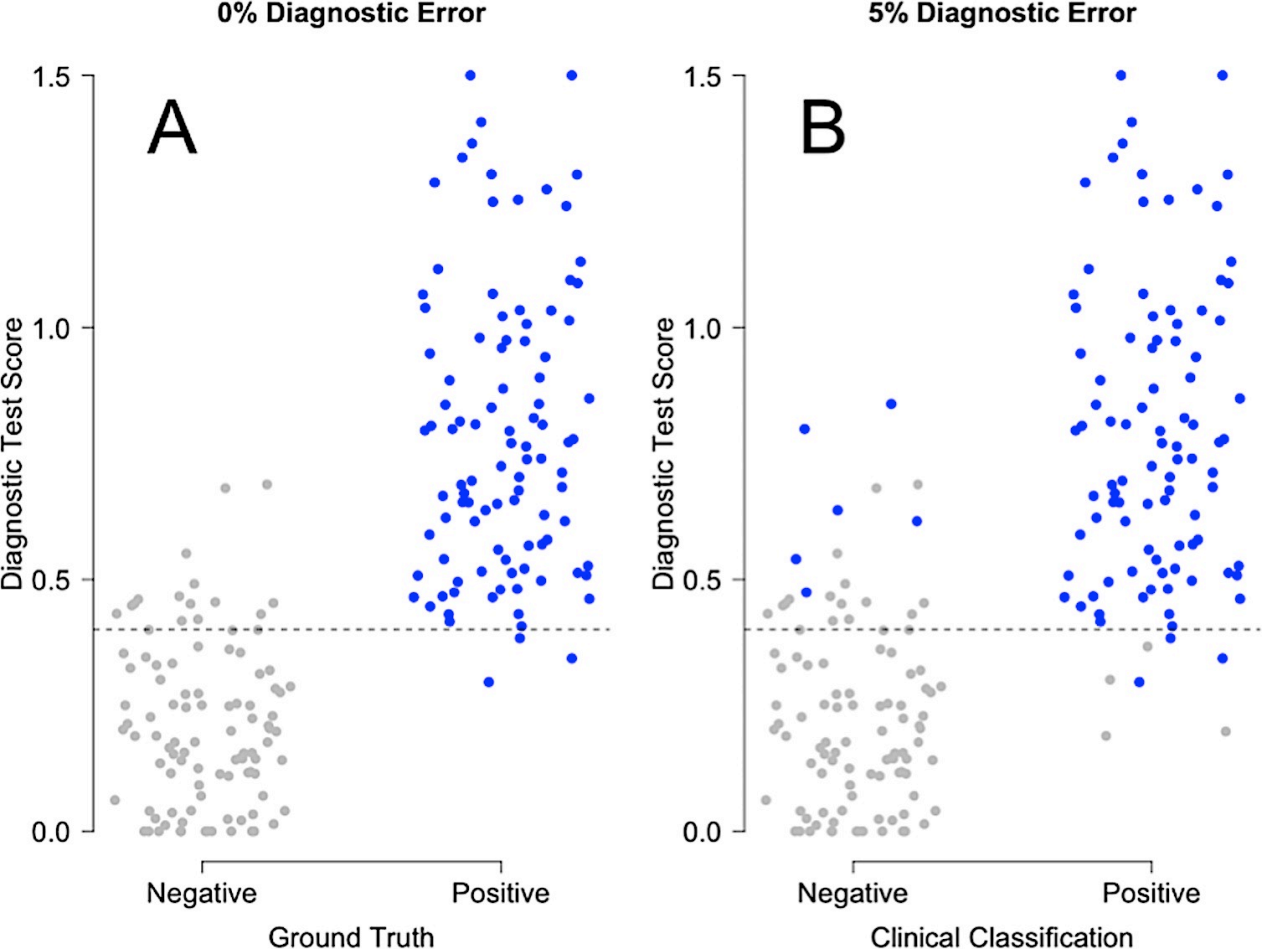
Example of the effect of misclassification by a comparator, on the apparent performance of a diagnostic test. A total of 100 Ground Truth negative patients and 100 Ground Truth positive patients were considered. In Panel A, there is no error in patient classification (i.e. the comparator is perfectly concordant with the Ground Truth). In Panel B, a random 5% of the comparator’s classifications are assumed to diverge incorrectly from the Ground Truth. The difference in the distribution of test scores (y-axis) between the panels of this figure results in significant underestimates of diagnostic performance as shown in Table 1.

Fig 2 shows graphically the effect of misclassification by the comparator on the interpretation of diagnostic test performance. This figure was generated from a simulation with 100 Ground Truth negative samples and 100 Ground Truth positive samples. Panel A shows the true test performance (0% comparator misclassification), while Panel B shows the effect of randomly injecting 5% misclassification into the comparator calls. The quantitative results from this particular simulation are compiled in Table 1. To assess the significance of the apparent differences in misclassification rates suggested by this table, we conducted a further investigation in which the number of simulated samples (trial size) was varied (S5 Supporting Information, “Decrease in apparent performance of index test, with 5% noise injected into comparator”). As expected, we found all the confidence intervals to shrink with increasing trial size. These results demonstrate the generality that for AUC, sensitivity/PPA, specificity/NPA, PPV and NPV, any degree of misclassification will lead to underestimates of true performance which can be detected if the trial is large enough and if the Ground Truth is known.

**Table 1:** Effect of uncertainty in the comparator on estimates of test performance.

Parameter	0% Misclassification Rate in the Comparator	5% Misclassification Rate in the Comparator
**AUC**	0.977 (0.960–0.993)	0.940 (0.910–0.971)
**Sensitivity/PPA**	0.970 (0.915–0.994)	0.929 (0.858–0.971)
**Specificity/NPA**	0.850 (0.765–0.914)	0.794 (0.703–0.868)
**PPV**	0.866 (0.789–0.923)	0.812 (0.728–0.880)
**NPV**	0.966 (0.904–0.993)	0.920 (0.843–0.967)

This table corresponds to Fig 2. A total of 100 Ground Truth negative patients and 100 Ground Truth positive patients were considered. The 95% confidence intervals about the medians were computed by resampling and are given within the parentheses.

**Fig 3:**
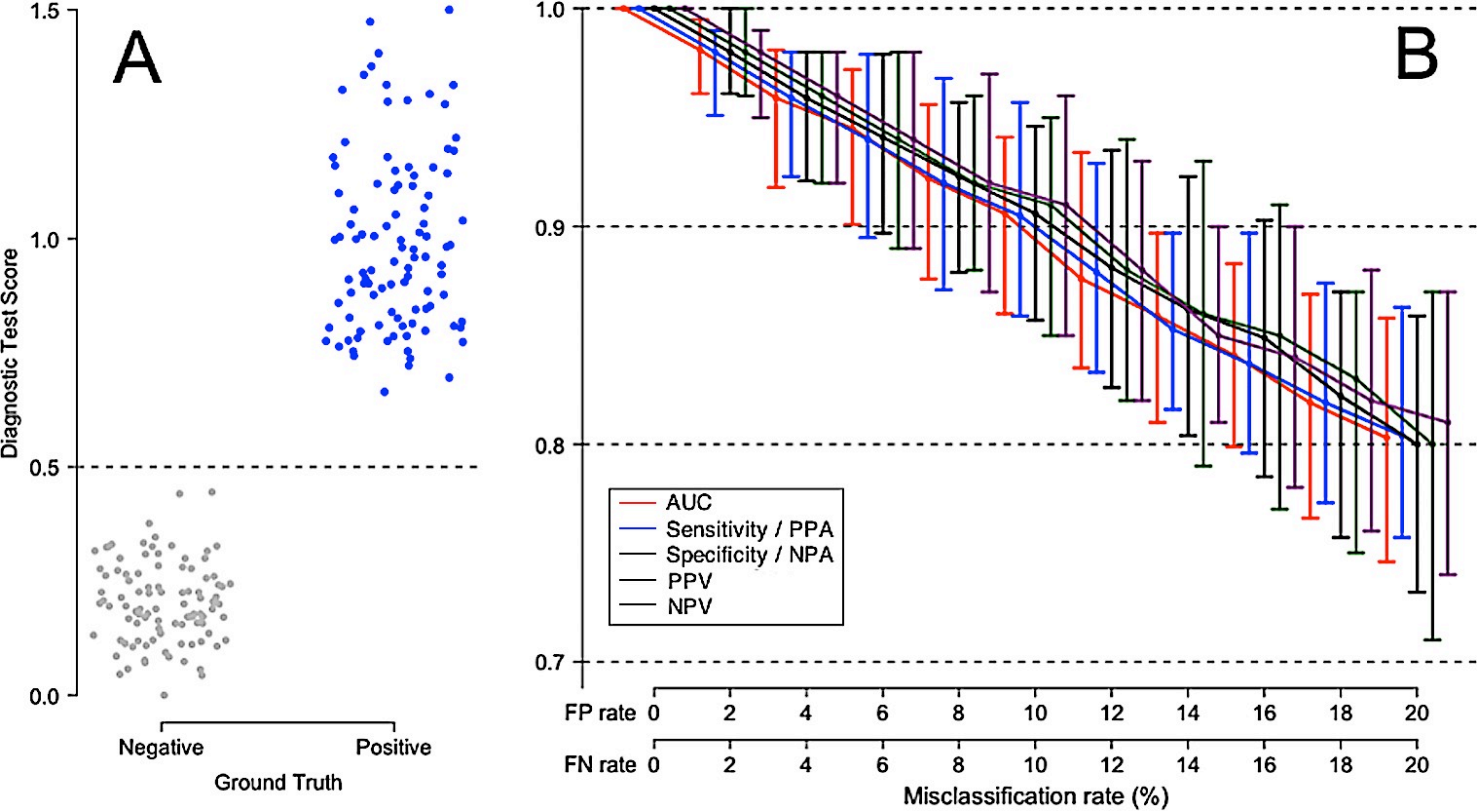
Degradation of apparent performance of a perfect diagnostic test, as a function of error in the comparator. In this scenario, Ground Truth positive patients and Ground Truth negative patients are equally likely to be misclassified by the comparator. (A) Comparator with no classification error, perfectly representing the Ground Truth for 100 negative patients and 100 positive patients. (B) Apparent performance of diagnostic test, as a function of the misclassification rate of the comparator. The error bars describe 95% empirical confidence intervals about medians, computed over 100 simulation cycles. True test performance is indicated when the FP and FN rates are each 0%. The terms Sensitivity and Specificity are appropriate when there is no misclassification in the comparator (FP rate = FN rate = 0%). The terms Positive Percent Agreement (PPA) and Negative Percent Agreement (NPA) should be used in place of Sensitivity and Specificity, respectively, when the comparator is known to contain uncertainty.

Fig 3 shows the effect of false positive and false negative comparator misclassifications on the apparent performance of a perfect test. In this simulation, there is no overlap between Ground Truth negative and Ground Truth positive patients. The test is assumed 100% accurate, so the reduced test performance values shown under various comparator misclassification rates are purely a result of uncertainty in the comparator. Varying the comparator misclassification rate between 0% and 20% results in a monotonic drop in AUC and other performance measures. Fig 3 also illustrates the point that the observed decrease in apparent test performance due to comparator noise can be expressed relative to the maximum possible test performance in the absence of comparator noise.

It appears from these simulations that once the comparator misclassification rates are in excess of about 5%, all test performance measures are significantly under-estimated and therefore should not be reported without acknowledging this effect. This figure also shows that when classification uncertainty is present in the comparator, any calculated performance measure will fall randomly within a range represented in Fig 3 by confidence intervals. As the comparator uncertainty increases, in addition to the general downward trend on the median apparent test performance value, the apparent test performance values will vary randomly within increasingly large ranges, as represented by increasingly wide confidence intervals.

Fig S6.1, presented in S6 Supporting Information (“Unequal FP and FN rates”), displays a modification of this effect, in which the false positive rate is twice as high as the false negative rate (Panel B of this figure). This situation could occur, for example, in the diagnosis of a serious infectious disease for which a treatment exists, where a clinician typically will ‘err on the side of caution’ in classifying patients, under the assumption that it is better to over-treat with side effects than under-treat with serious consequences (false positives being considered less risky than false negatives). Fig S6.2, presented in S6 Supporting Information (“Unequal FP and FN rates”), displays the complementary scenario in which the false negative rate is twice as high as the false positive rate. Certain types of tests, for example home pregnancy tests, are known to suffer from high false negative rates [26,27].

**Fig 4:**
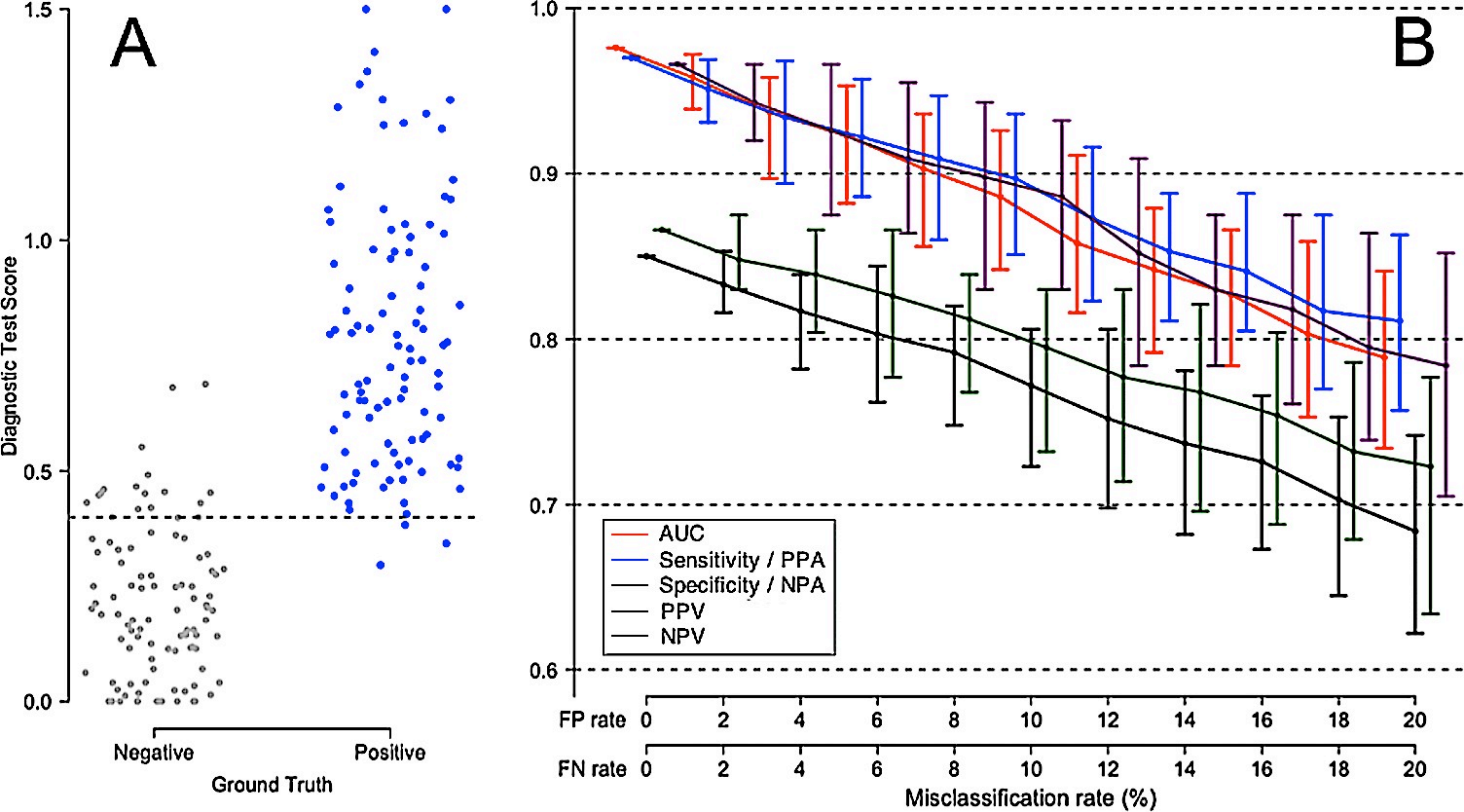
Degradation of apparent performance of a perfect diagnostic test, as a function of error in the comparator. (A) Representation of Ground Truth for 100 negative patients (grey points) and 100 positive patients (blue points). A slight overlap between Ground Truth negative and Ground Truth positive distributions is assumed, leading to AUC 0.98 with the Ground Truth as reference. (B) Apparent performance of a perfect diagnostic test, as a function of the misclassification rate of the comparator. The error bars describe 95% empirical confidence intervals about medians, computed over 100 simulation cycles. True test performance is indicated when the FP and FN rates are each 0%. The terms Sensitivity and Specificity are appropriate when there is no misclassification in the comparator (FP rate = FN rate = 0%). The terms Positive Percent Agreement (PPA) and Negative Percent Agreement (NPA) should be used in place of Sensitivity and Specificity, respectively, when the comparator is known to contain uncertainty.

Fig 4 displays a less idealized diagnostic scenario, in which there is some small degree of overlap between Ground Truth negative and Ground Truth positive patients. We consider such a typical high-performing test, and estimate the degradation of apparent test performance under conditions of increasing comparator uncertainty. Panel A shows the distribution of test results against the Ground Truth. Panel B shows the expected decrease in all test performance parameters, as a monotonic function of increasing comparator uncertainty. Note the generally worse apparent test performance in Fig 4 at all levels of comparator misclassification, as compared to Fig 3 in which Ground Truth negative and Ground Truth positive patients display no overlap in diagnostic test scores.

**Fig 5:**
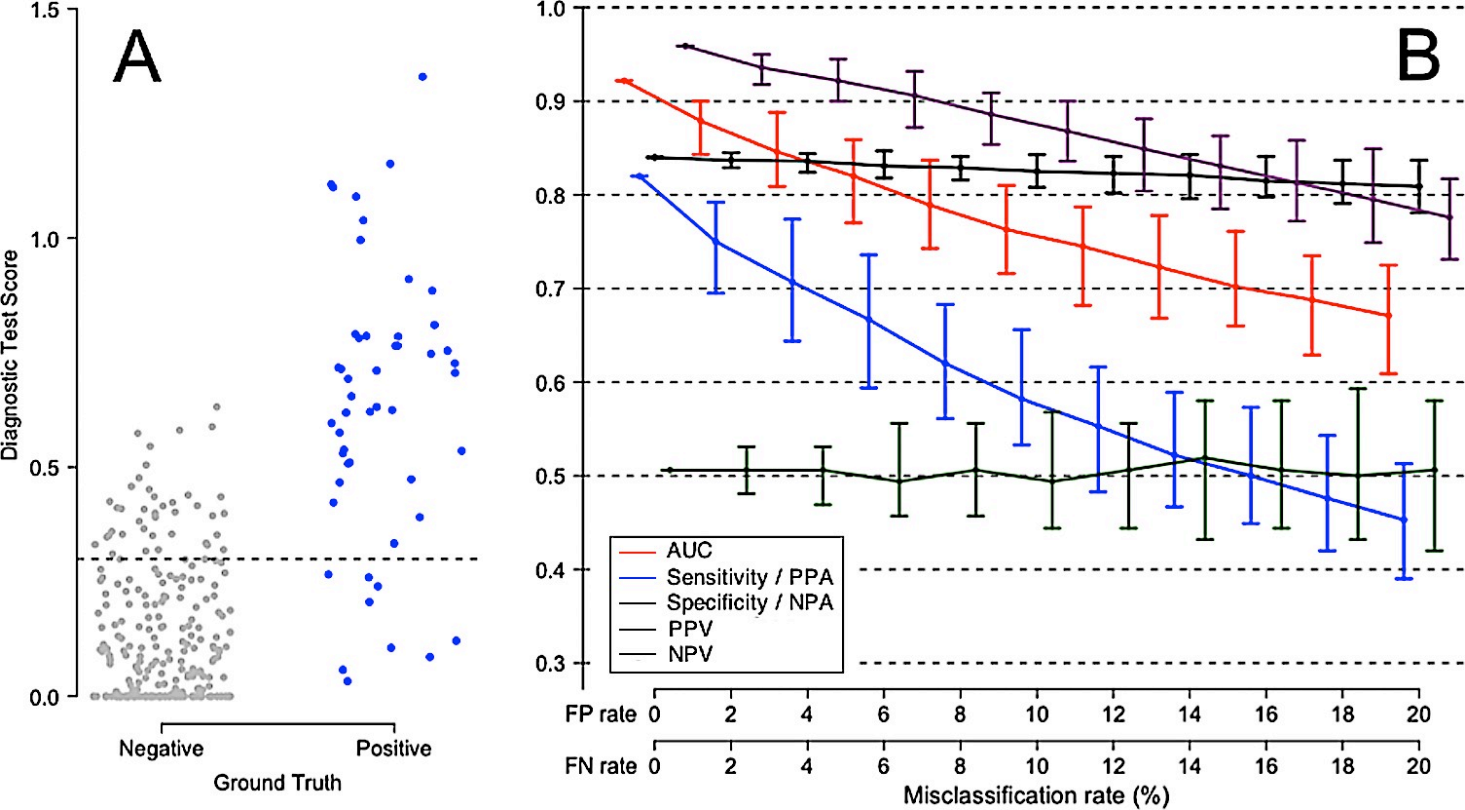
A simulated inaccurate screening test in a moderately low prevalence setting. In this scenario, ground truth positive patients are equally likely to be misclassified as ground truth negative patients. (A) Representation of Ground Truth for 250 negative patients, and 50 positive patients, with significant overlap between the positive and negative ground truth distributions. (B) Apparent performance of diagnostic test, as a function of the misclassification rate of the comparator. The error bars describe 95% empirical confidence intervals about medians, computed over 100 simulation cycles. True test performance is indicated when the FP and FN rates are each 0%. The terms Sensitivity and Specificity are appropriate when there is no misclassification in the comparator (FP rate = FN rate = 0%). The terms Positive Percent Agreement (PPA) and Negative Percent Agreement (NPA) should be used in place of Sensitivity and Specificity, respectively, when the comparator is known to contain uncertainty.

Fig 5 simulates a screening test in a low prevalence setting, in which Ground Truth negatives are significantly more prevalent than Ground Truth positives. An example of such a scenario is found in screening for cervical cancer by Pap smear cytology, in which significant false positive rates (cellular abnormalities of unknown significance) may be anticipated, and in which positive test results do not necessarily confer high confidence regarding the presence of high level disease [28,29].

**Fig 6:**
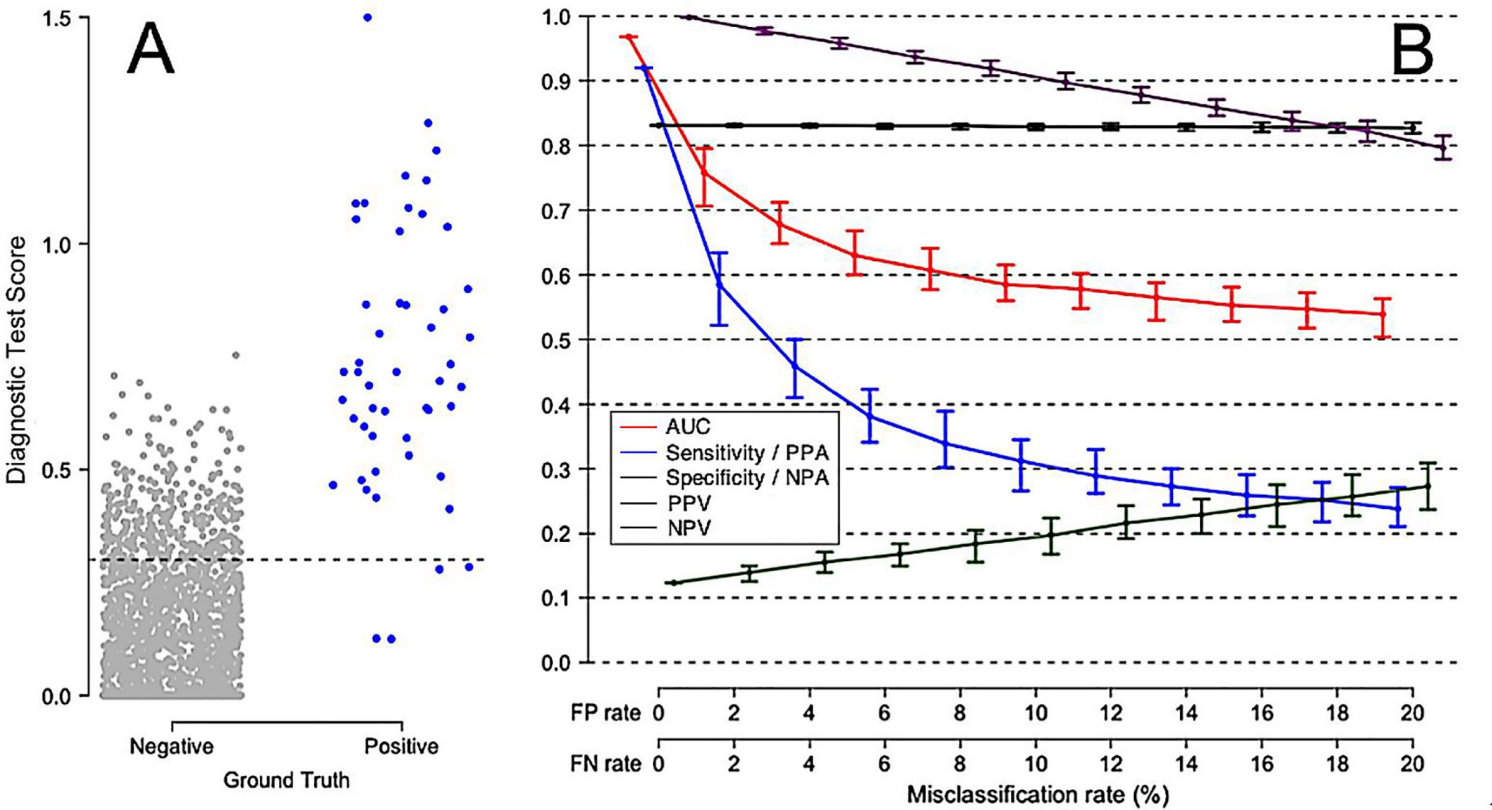
A simulated screening test in a low prevalence setting, for example for a relatively uncommon infectious disease. In this scenario ground truth positive patients are equally likely to be misclassified as negative patients (A) Representation of Ground Truth for 1950 negative patients, and 50 positive patients. with some overlap between the positive and negative ground truth distributions. (B) Apparent performance of diagnostic test, as a function of the misclassification rate of the comparator. The error bars describe 95% empirical confidence intervals about medians, computed over 100 simulation cycles. True test performance is indicated when the FP and FN rates are each 0%. The terms Sensitivity and Specificity are appropriate when there is no misclassification in the comparator (FP rate = FN rate = 0%). The terms Positive Percent Agreement (PPA) and Negative Percent Agreement (NPA) should be used in place of Sensitivity and Specificity, respectively, when the comparator is known to contain uncertainty.

Fig 6 simulates the effect of comparator uncertainty in a screening test scenario of even lower disease prevalence, for example an epidemiological setting [30,31].

### Actual data

We next turn to the analysis of a real dataset, collected during a clinical validation study of a novel diagnostic test for sepsis [25]. The test was designed to discriminate infection-induced sepsis from non-infectious systemic inflammatory response syndrome (SIRS) in adult critical care patients, and was validated using a cohort of 447 patients from seven sites in the USA and one site in the Netherlands. In the validation study, the comparator consisted of retrospective physician diagnosis (RPD) by a panel of three independent expert clinicians, leading to either the unanimous, consensus, or forced classification of patients as having either sepsis or SIRS.

**Fig 7:**
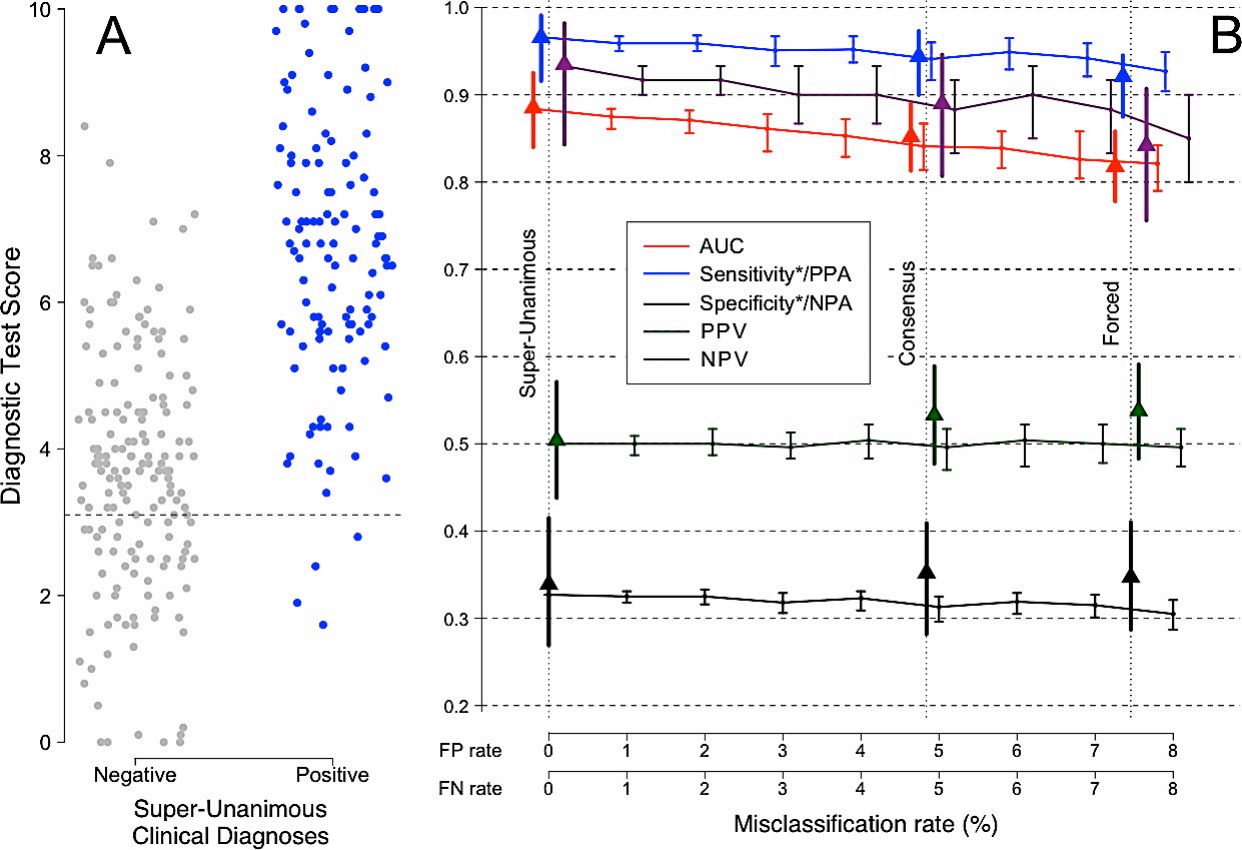
(A) Real data from a clinical trial for a new sepsis diagnostic test, conducted over 8 sites in the USA and Netherlands [25]. (B) The apparent performance of the test (y axis) decreases as uncertainty is introduced into the comparator (x axis). 95% confidence intervals are shown. The difference between the apparent test performance at a given comparator misclassification rate and at a comparator misclassification rate of zero indicates the degree of underestimation of true test performance due to uncertainty in the comparator. The vertical lines mark the observed misclassification rates for various patient subsets within the same trial, as described in the text. Misclassification rates are based on quantifying the discordance between independent expert opinions. Solid triangles show the observed measurements for the trial for each of these groups without correction for comparator uncertainty. Sensitivity/PPA and Specificity/NPA are each marked with an asterisk (*) to emphasize that these measures assume no misclassification in the comparator. Positive Percent Agreement (PPA) and Negative Percent Agreement (NPA) are the correct terms to use, when the comparator is known to contain uncertainty as in this case.

Fig 7, Panel A shows the distribution of test scores for the Super-Unanimous subset of 290 patients (119 sepsis, 171 SIRS), defined as those patients who were classified as either sepsis or SIRS by all three of the external expert panelists and also by the study investigators at the clinical sites where the patients were recruited. As stated previously, these patients represent the stratum of the trial cohort with the lowest expected probability of error in the comparator. Panel B shows the calculated estimates of performance (AUC, sensitivity/PPA, specificity/NPA, PPV, NPV) as an increasing amount of uncertainty is introduced into the comparator. The false positive (FP) and false negative (FN) rates for the Super-Unanimous subset are assumed to be zero, as shown by the leftmost vertical dotted line of panel B. For the Consensus subset, the observed misclassification rates were 4.9% FP and 4.7% FN, which correspond to an injection of about 4.84% random misclassifications into the Super-Unanimous subset. For the Forced subset, the observed misclassification rates were 6.1% FP and 9.0% FN, which correspond to an injection of about 7.46% random misclassifications into the Super-Unanimous subset. In panel B, the triangles indicate the calculated values of the performance parameters (AUC, sensitivity/PPA, specificity/NPA, PPV, NPV), after injection of the stated amounts of uncertainty (random misclassification noise) into the comparator.

We simulated the effects of comparator uncertainty by starting with the Super-Unanimous classifications (assumed to be error free), injecting noise (uncertainty) into the underlying comparator (randomly sampled from the empirical noise distribution), and calculating the resultant increase in apparent FP and FN rates. As the comparator misclassification rate increased, the apparent performance of the new diagnostic test declined, consistent with the earlier simulation studies shown in Figs 2–6. Specifically, as the comparator noise level was increased, there was a corresponding decrease in AUC. When expressed in terms of relative error (relative error = (1-AUC)/(1-AUC_0_) where AUC_0_ = AUC at zero misclassification rate), we found that each 1% increase in comparator noise produced an approximately 9% increase in relative error. With the injection of 4.8% misclassifications into the Super-Unanimous comparator, the simulation contained as much noise as was observed in the Consensus subset of the actual clinical trial data. Similarly, with the injection of 7.5% misclassifications, the simulation contained as much noise as was observed for the Forced group, as shown in Table 2. The comparison between simulated (predicted) and observed diagnostic test performance is shown in Table 2.

**Table 2:** Testing the models: Simulated vs. observed effect of comparator noise on test performance.

Parameter	Super-Unanimous (N = 290) FP Rate = 0.0% FN Rate = 0.0%		Consensus (N = 410) FP rate = 4.9% FN rate = 4.7%		Forced (N = 447) FP rate = 6.1% FN rate = 9.0%	
	No misclassifications injected into Super-Unanimous	Observed	4.84% misclassifications injected into Super-Unanimous	Observed	7.46% misclassifications injected into Super-Unanimous	Observed
**AUC**	0.887 (0.848–0.926)	0.887 (0.848–0.926)	0.839 (0.805–0.863)	0.852 (0.814–0.890)	0.815 (0.777–0.846)	0.818 (0.778–0.858)
**Sensitivity** **/ PPA**	0.967 (0.918–0.991)	0.967 (0.918–0.991)	0.950 (0.932–0.966)	0.944 (0.900–0.973)	0.946 (0.926–0.965)	0.921 (0.875–0.954)
**Specificity** **/ NPA**	0.339 (0.269–0.415)	0.339 (0.269–0.415)	0.322 (0.307–0.337)	0.352 (0.291–0.418)	0.313 (0.297–0.329)	0.347 (0.287–0.410)
**PPV**	0.509 (0.442–0.575)	0.509 (0.442–0.575)	0.491 (0.461–0.513)	0.533 (0.477–0.589)	0.465 (0.435–0.500)	0.538 (0.483–0.591)
**NPV**	0.936 (0.843–0.982)	0.936 (0.843–0.982)	0.903 (0.871–0.935)	0.890 (0.807–0.946)	0.903 (0.871–0.935)	0.842 (0.756–0.907)

Clinical trial data from Miller et al. [25] were used. The 95% confidence intervals are given within the parentheses. The following tradeoff for a binary test is reflected in the data: a high value of sensitivity/PPA will imply a low value of specificity/NPA.

Of the 447 patients in the trial, 93 were diagnosed by consensus RPD as having pneumonia or lower respiratory tract infections (LRTI). With respect to the secondary diagnosis of sepsis vs. SIRS, very high levels of disagreement (uncertainty) by the expert panelists were found for this subset of patients. In only 45/93 (48%) of these cases did all three external panelists agree on the diagnosis of sepsis or SIRS. A further indication of the difficulty of diagnosing pneumonia/LRTI patients as having sepsis or SIRS came from an examination of the 37/447 patients classified as indeterminate by the consensus RPD of the three external panelists. Of these 37 patients, 20 (54%) were diagnosed with pneumonia/LRTI (Table 3). The misclassification rates for this sub-population were calculated to be 17.5% FP, 13.7% FN, 14.4% overall.

**Table 3:** Classification of patients in a trial on a new sepsis diagnostic test.

Comparator	Condition	SIRS	Indeterminate	Sepsis
**A. Unanimous RPD** **(N = 315)**	All other conditions	169 (53.6%)	0 (0.0%)	100 (31.7%)
	Pneumonia / LRTI	4 (1.3%)	1 (0.3%)	41 (13.0%)
**B. Super-Unanimous RPD (N = 290)**	All other conditions	168 (57.9%)	0 (0.0%)	90 (31.0%)
	Pneumonia / LRTI	3 (1.0%)	0 (0.0%)	29 (10.0%)
**C. Consensus RPD** **(N = 447)**	All other conditions	221 (49.4%)	17 (3.8%)	116 (26.0%)
	Pneumonia / LRTI	9 (2.0%)	20 (4.5%)	64 (14.3%)

The trial is described in Miller et al. [25]. (A) Unanimous RPD, in which all three external panelists agreed on diagnosis of sepsis, SIRS, or indeterminate. (B) Super-Unanimous RPD, in which all three external panelists, and also the study investigators at the clinical site of origin, agreed on the diagnosis of sepsis, SIRS, or indeterminate. (C) Consensus RPD, in which two or all three external panelists agreed on diagnosis of sepsis, SIRS, or indeterminate.

**Fig 8:**
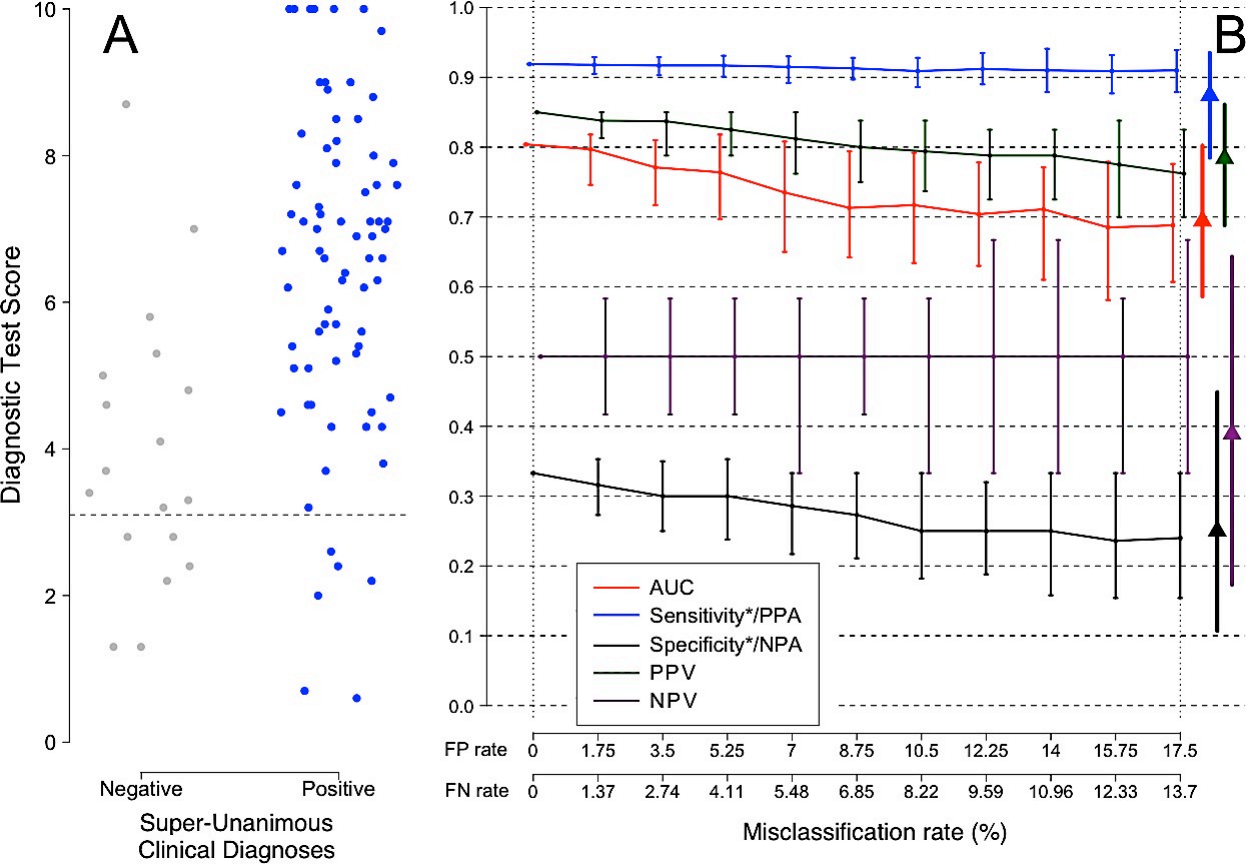
(A) Subset of pneumonia/LRTI-specific data (N = 93) from a clinical trial for a new sepsis diagnostic test, conducted over 8 sites in the USA and Netherlands [25]. (B) The apparent performance of the test (y axis) decreases as uncertainty is introduced into the comparator (x axis). 95% confidence intervals are shown. The difference between the apparent test performance at a given comparator misclassification rate and at a comparator misclassification rate of zero indicates the degree of underestimation of true test performance due to uncertainty in the comparator. Solid triangles show the observed measurements for the trial for each of these groups without correction for comparator uncertainty. Misclassification rates are based on quantifying the discordance between independent expert opinions. Sensitivity/PPA and Specificity/NPA are each marked with an asterisk (*) to emphasize that these measures assume no misclassification in the comparator. Positive Percent Agreement (PPA) and Negative Percent Agreement (NPA) are the correct terms to use, when the comparator is known to contain uncertainty as in this case.

For the patients with pneumonia or LRTI, the level of uncertainty in the comparator was roughly double that of the non-pneumonia patients. The two groups also differed in the prevalence of sepsis (see Table 3, p <0.001), the pattern of physician discordance in classification, and the distribution of SeptiCyte LAB test scores. Fig 8, Panel A shows a subset of super-unanimously classified sepsis and SIRS patients from the complete trial population, selected to match the sepsis prevalence and test score distribution of pneumonia/LRTI patients. Fig 8, Panel B shows the effects of increasing the comparator misclassification rate on the performance parameters for this patient subset. The triangles in this panel signify the parameter values observed for the pneumonia/LRTI patients. The triangles have been placed at a position along the x-axis (17.5% FPR, 13.7% FNR) that is appropriate for the pneumonia/LRTI patient group, as inferred from the measured discordance in the comparator diagnoses for this group (see S2–S4 Supporting Information).

Regarding the 45 pneumonia/LRTI patients who were diagnosed by all three expert panelists to be either positive or negative for sepsis, we observed no significant difference in test performance between this patient subset and the subset of all patients with Super-Unanimous diagnoses across the trial as a whole (Table 3). Simulations in which a weighted average 14.4% misclassification rate for pneumonia/LRTI patients was introduced into the Super-Unanimous RPD comparator led to predicted underestimates of performance that were well aligned with the observed measurements of test performance (Fig 8, Panel B, triangles and Table 4).

**Table 4:** Demonstration of the effect of comparator uncertainty on estimates of test performance, for the pneumonia/LRTI patient subset.

Parameter	Minimal uncertainty in comparator classification		17.5% False Positive rate 13.7% False Negative rate (due to comparator uncertainty)	
	A. All conditions (N = 290)	B. Pneumonia /LRTI (N = 45)	C. Introduced to Col A. (N = 290)	D. Observed in pneumonia/LRTI (N = 45)
**AUC**	0.887 (0.848–0.926)	0.87 (0.71–1.00)	0.679 (0.576–0.723)	0.67 (0.64–0.72)
**Sensitivity/PPA**	0.967 (0.918–0.991)	0.97 (0.84–1.00)	0.909 (0.783–0.975)	0.89 (0.85–0.92)
**Specificity/NPA**	0.339 (0.269–0.415)	0 (0.00–0.52)	0.274 (0.169–0.402)	0.28 (0.25–0.30)
**PPV**	0.509 (0.442–0.575)	0.86 (0.71–0.96)	0.471 (0.361–0.582)	0.46 (0.42–0.50)
**NPV**	0.935 (0.843–0.982)	0 (0.00–0.98)	0.810 (0.581–0.946)	0.79 (0.71–0.85)

The clinical trial of a new sepsis test is described in the publication of Miller et al. [25]. Column A: Test performance for the Super-Unanimous subset of patients (N = 290) who received unanimous diagnoses of sepsis or SIRS by three external expert panelists, and who were also assigned the same diagnosis by the study investigators at the clinical sites where the patients originated. Column B: Test performance for the subset of pneumonia/LRTI patients from column A. Column C: Based on the uncertainty distribution observed in the trial, a comparator misclassification rate of 17.54% FP, 13.73% FN, 14.4% overall was estimated for the pneumonia/LRTI patient subset, and introduced into the simulation. The simulation was repeated 100 times with median and 95% CIs indicated. Column D: Observed performance metrics for the pneumonia / LRTI subset of the Super-Unanimous population (Column A). The following tradeoff for a binary test is reflected in the data: a high value of sensitivity/PPA will imply a low value of specificity/NPA.

### The unattainability of a perfect test

Additional simulations showed that it is extremely unlikely for any test, even a perfect test, to achieve very high performance in a diagnostic evaluation trial, when even a small amount of uncertainty is present in the comparator against which the test is being evaluated. For example, as shown in S7 Supporting Information (“Very high performance tests”), if 99% PPA (sensitivity) or NPA (specificity) is required in a diagnostic evaluation trial, then a modest 5% patient misclassification rate in the comparator will lead to rejection of the perfect diagnostic test with a probability greater than 99.999%. Thus a specific numerical requirement for test performance, especially a very high performance requirement such as 99% PPA (sensitivity), can only be meaningfully discussed if all classification uncertainty in a trial is either ruled out or characterized, and the measured test performance interpreted with respect to the theoretical limits imposed by comparator uncertainty.

## Discussion

The performance of any diagnostic test must be evaluated in reference to a comparator. The presence of noise (classification uncertainty) in the comparator is therefore an important confounding factor to consider in the interpretation of the performance of diagnostic tests. As increasing amounts of noise are introduced into the comparator, the apparent performance of the diagnostic test (relative to the comparator) will decline in a concomitant fashion. This is consistent with the general expectation that the addition of randomness into a method for analyzing a test’s performance should drag any performance indicator down towards a limiting minimum value (for example 0.50 for AUC). While this confounding factor was first identified over 30 years ago [12], it has received renewed attention in the 2015 STARD standard for diagnostic reporting [32,33] under checklist item #15 (“How indeterminate index test or comparator results were handled”).

In the analysis of real world data from a clinical trial on a new sepsis test [25], significant differences in apparent test performance were measured in different patient subsets. One interpretation is that the actual performance of the test varied, depending on the patient subset being considered. Although genuine differences in test performance may indeed exist between patient subsets, an alternative explanation is suggested by two key results: 1) the correspondence between the observed performance and the predicted performance as a function of comparator noise level; 2) the lack of a significant difference in test performance between patient subsets, when comparator noise was removed by excluding patients with uncertainty in the comparator diagnosis. These results support the hypothesis that the diagnostic test performed equally well across all patient subsets, but varying levels of comparator noise led to the appearance of diagnostic test performance differences.

Our simulations show, perhaps counter-intuitively, that increasing uncertainty in the comparator can sometimes have a non-linear effect on performance (as measured by different performance indicators such as AUC, PPA, NPA). The non-linearity is most pronounced when disease prevalence is either high or low (see for example Fig 6). This non-linearity increases the difficulty of intuiting the effect of uncertainty on evaluating diagnostic performance, because linear extrapolation under these conditions may lead to erroneous conclusions. The use of a simulation tool, such as the one associated with this paper, may be the only realistic way for non-statistical experts to verify that the level of uncertainty measured in their trial does not invalidate their conclusions.

An upper bound on the apparent performance of a test will be imposed by the presence of noise in the comparator. For example, consider a misclassification rate of 10% in a comparator. This would impose a maximum theoretical AUC of 0.90 (95% CI 0.86–0.94) for any test, including a perfect test, that is measured against this comparator, as shown in Fig 3. If using this comparator a new diagnostic test measures an apparent AUC of 0.89 (95% CI 0.87–0.91), then the test would be statistically indistinguishable from perfect, and should be reported as such. To report an AUC of 0.89 for this new test without this important qualifier would be misleading. The simulation tool associated with this paper can be used to generate theoretical maximum limits on performance estimates under conditions of comparator uncertainty, against which trial results can be assessed in the context of their proximity to theoretical perfection under these conditions.

Our simulations also reveal that for tests requiring high accuracy, the presence of uncertainty in the comparator will be highly detrimental. For example, degradation of apparent test performance (AUC) from a true performance level of 0.97 AUC to a measured performance level of 0.93 AUC, while only -0.04 AUC units in absolute terms, in fact represents more than a doubling of the apparent error rate of the test (from 3% to 7%), due solely to the comparator noise effect. This could lead to a decision not to adopt a test in the clinic because the test performance is (erroneously) assumed to be inadequate.

For tests requiring even higher accuracy, for example 99% sensitivity or negative predictive value, extreme caution must be exercised in trial interpretation if even small amounts of uncertainty may be present in the comparator. In these cases, an apparently reasonable requirement for robust test performance will result in the rejection of even a perfect test, in almost all cases, due to failure to account for the effects demonstrated in this paper. Stakeholders interested in ensuring very high performance (i.e. 99% sensitivity or NPV) must bear in mind that such high performance characteristics can only be practically demonstrated with respect to a nearly flawless comparator method. In the absence of a nearly flawless comparator method, it will not be possible to validate such high test performance characteristics, and attempts to do so will likely result in underestimation of candidate test performance. The impact of an imperfect comparator on very high performance tests is analyzed quantitatively in S7 Supporting Information (“Very high performance tests”).

Generally, it can be seen from both the simulations and our actual data that a 5% or greater misclassification rate in the comparator may result in significant underestimates of test performance, which could in turn have significant consequences, e.g. in a clinical trial. If there is reason to suspect a misclassification rate above this limit, it is advisable (in accord with STARD criterion #15) to report the comparator uncertainty together with the estimated performance of the new test. The estimated test performance should be reported relative to the expected performance of a perfect test under the prevailing conditions of uncertainty, which can be estimated with the simulation tool that accompanies this paper. At minimum, the amount of comparator uncertainty should be measured or described so that its effect can be bounded or incorporated into the interpretation of the data. Without taking classification uncertainty into account, researchers risk arriving at false or biased conclusions.

## Conclusions

This study has shown, with both simulated and real data, that noise (classification uncertainty) in a comparator will exert a significant downward effect on the apparent performance of any new test under evaluation. The common condition of uncertainty in clinical trial classifications, combined with the knowledge that very few such trials measure and account for this uncertainty, could potentially undermine the conclusions drawn from such trials, if the effect is not corrected for. If there is reason to suspect a significant amount of classification uncertainty in the comparator, it is advisable to report the comparator uncertainty together with the estimated performance of the new test. We provide an online simulation tool to allow researchers to explore the effect of comparator noise, using their own trial parameters: https://imperfect-gold-standard.shinyapps.io/classification-noise/. The source code for the simulation tool has been made publicly available: https://github.com/ksny/Imperfect-Gold-Standard.

## Supporting information

S1 Supporting Information Example of reference bias. The example shown here leads to over-estimation of test performance.
(PDF)

S2 Supporting Information Method to estimate the confidence of patient classifications by an expert panel comparator. In the clinical trial described by Miller et al. [25], three clinicians provided independent patient diagnoses while blinded to the diagnoses of each other. For an individual clinician’s diagnosis regarding the presence of systemic infection in an individual patient, a classification of ‘No’ carried a probability of systemic infection of zero, ‘Yes’ carried a probability of one, and ‘Indeterminate’ carried a probability of one half. The overall infection probability was calculated as a simple average of the three input values.
(PDF)

S3 Supporting Information Weighting for introduced misclassification events. Using the dataset of Miller et al. [25] and the method described in S2 Supporting information, each patient receives a probabilistic assessment of his or her systemic infection status. Samples are then selected, on the basis of the observed uncertainty distribution. This selection process reflects the expectation that patients with more certainty in classification are less likely to be misclassified. The selected samples are then relabeled with the opposite status indicator.
(PDF)

S4 Supporting Information Calculating misclassification rates, based on patient confidence values. The overall expected total misclassification rate is the FP rate applied to the negative patients plus the FN rate applied to the positive patients.
(PDF)

S5 Supporting Information Decrease in apparent performance of index test, with 5% noise injected into comparator. A set of simulation runs was conducted to further explore the effect, on apparent performance of an index test, of 5% noise injected into the comparator.
(PDF)

S6 Supporting Information Unequal FP and FN rates. The examples show degradation of apparent performance of a diagnostic test as a function of noise in the comparator, when FP and FN rates are not equal.
(PDF)

S7 Supporting Information Very high performance tests. The analysis shows that the goal of achieving a very high diagnostic test performance, such as a 99% sensitivity, can be rendered nearly unreachable, if the comparator diagnosis contains even small amounts of uncertainty.
(PDF)

## Abbreviations

ROC: receiver operating characteristic
AUC: area under ROC curve
CI: confidence interval
LRTI: lower respiratory tract infection
NPA: negative percent agreement
NPV: negative predictive value
PPA: positive percent agreement
PPV: positive predictive value
ROC: receiver operating characteristic
RPD: retrospective physician diagnosis
SIRS: systemic inflammatory response syndrome
