# Correction: The effect of uncertainty in patient classification on diagnostic performance estimations

**DOI:** 10.1371/journal.pone.0218492

**Published:** 2019-06-11

**Authors:** Leo C. McHugh, Kevin Snyder, Thomas D. Yager

The following information is missing from the Competing Interests section: This publication reflects the views of the authors and should not be construed to represent the FDA’s views or policies.
