# Supplementary material for: The effect of uncertainty in patient classification on diagnostic performance estimations
Source: PLoS One. 2019 May 22;14(5):e0217146. doi: 10.1371/journal.pone.0217146 (PMC6530857; doi:10.1371/journal.pone.0217146)
Supplement: S1 Supporting Information — The example shown here leads to over-estimation of test performance. (PDF) [file pone.0217146.s001.pdf]

S1 Supporting information: Example of reference bias

The presence of systemic infection (sepsis) is sometimes inferred through a combination of measurements of inflammation-related biomarkers [1-5]. However, conditions besides sepsis can cause inflammation, so diagnosic tests for sepsis that are based on inflammatory markers may give false positives due to other inflammatory conditions.

Suppose, for the sake of argument, that a comparator (reference test) for sepsis is defined in terms of such inflammation markers. If both the comparator and a new diagnostic test produce positive results in response to inflammation, then there will be a high correlation between the results of the comparator and the new test, even in cases where both are wrong. This leads to an inflation of the measured performance of the test relative to the comparator. This situation is shown in Fig. S1.1.

It is not difficult to imagine the converse situation, in which reference bias could lead to under-estimation of performance. This would occur if there was an anticorrelation in the response of the comparator and test under evaluation.

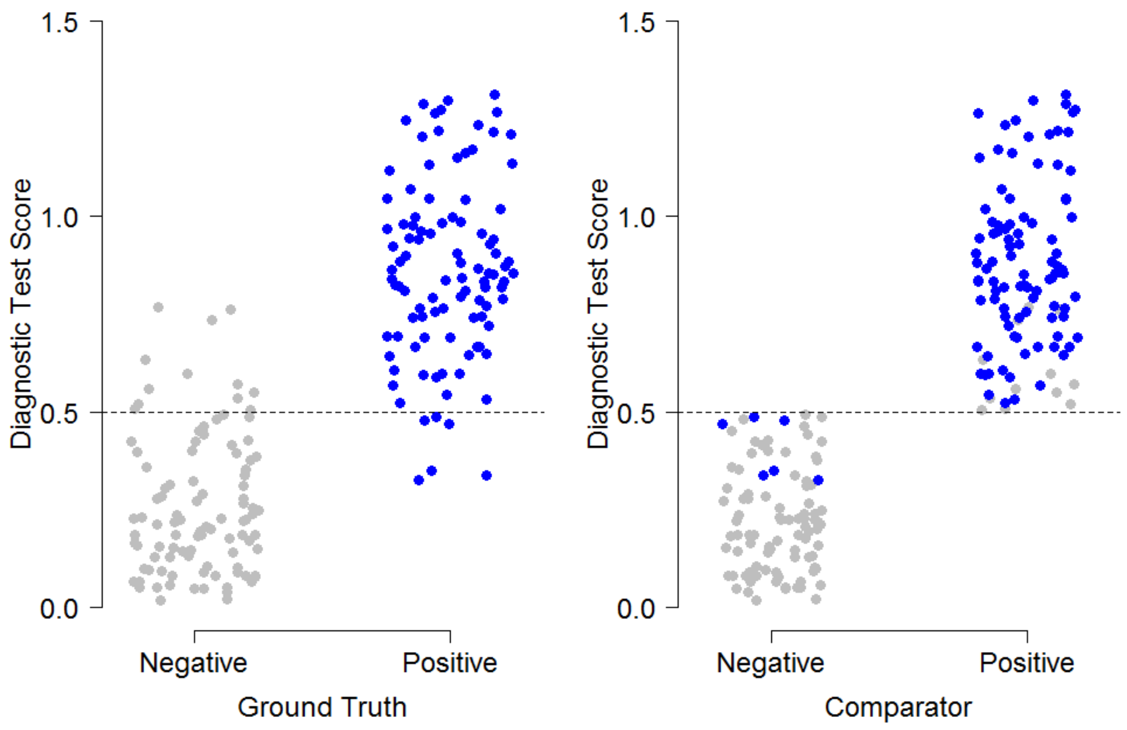

Fig. S1.1 Example of reference bias resulting in over-estimation of test performance. (A) Diagnostic test score (y-axis) compared to the Ground Truth, showing the true test performance. Grey points: 100 True Negatives at the level of Ground Truth. Blue Points: 100 True Positives at the level of Ground Truth. The test has AUC 0.976 using the Ground Truth as reference. (B) An example of where the comparator deviates from the Ground Truth in the same direction as the test, resulting in over-estimation of test performance. The test has AUC 1.00 using the biased comparator as reference.

References

1. Herzum I, Renz H. Inflammatory markers in SIRS, sepsis and septic shock. Curr Med Chem. 2008;15:581-587. doi: 10.2174/092986708783769704.

2. De Jong HK, van der Poll T, Wiersinga WJ. The systemic pro-inflammatory response in sepsis. J Innate Immun. 2010;2:422-430. doi: 10.1159/000316286.

3. Tsalik EL, Jaggers LB, Glickman SW, Langley RJ, van Velkinburgh JC, Park LP, et al. Discriminative value of inflammatory biomarkers for suspected sepsis. J Emerg Med. 2012;43:97-106. doi: 10.1016/j.jemermed.2011.05.072.

4. Machado JR, Soave DF, da Silva MV, de Menezes LB, Etchebehere RM, Monteiro ML, et al. Neonatal sepsis and inflammatory mediators. Mediators Inflamm. 2014; 269681. doi: 10.1155/2014/269681.

5. Talebi-Taher M, Babazadeh S, Barati M, Latifnia M. Serum inflammatory markers in the elderly: are they useful in differentiating sepsis from SIRS? Acta Med Iran. 2014;52:438-444.
