# Supplementary material for: The effect of uncertainty in patient classification on diagnostic performance estimations
Source: PLoS One. 2019 May 22;14(5):e0217146. doi: 10.1371/journal.pone.0217146 (PMC6530857; doi:10.1371/journal.pone.0217146)
Supplement: S2 Supporting Information — In the clinical trial described by Miller et al. [25], three clinicians provided independent patient diagnoses while blinded to the diagnoses of each other. For an individual clinician’s diagnosis regarding the presence of systemic infection in an individual patient, a classification of ‘No’ carried a probability of systemic infection of zero, ‘Yes’ carried a probability of one, and ‘Indeterminate’ carried a probability of one half. The overall infection probability was calculated as a simple average of the three input values. (PDF) [file pone.0217146.s002.pdf]

S2 Supporting information: Method to estimate the confidence of patient classifications by an expert panel comparator

In the clinical trial described by Miller et al. [1], three clinicians provided independent patient diagnoses while blinded to the diagnoses of each other. Each clinician classified patients as ‘No’, ‘Yes’ or ‘Indeterminate’ with respect to the presence of systemic infection (sepsis).

For an individual clinician’s diagnosis regarding the presence of systemic infection in an individual patient, a classification of ‘No’ carried a probability of systemic infection of zero, ‘Yes’ carried a probability of one, and ‘Indeterminate’ carried a probability of one half. The overall infection probability was calculated as a simple average of the three input values. For example, a patient with the individual classifications of ‘Indeterminate’, ’No’, ‘Yes’, was considered to have an overall probability of systemic infection of (0.5+0+1)/3 = 0.5.

An overall probability of zero meant that all clinicians agreed that systemic infection was not present, and an overall probability of one meant that all clinicians agreed that systemic infection was present. Values between these extremes represented some amount of uncertainty in patient classification.

References

1. Miller III RR, Lopansri BK, Burke JP, Levy M, Opal S, Rothman RE, et al. Validation of a Host Response Assay, Septicyte™ LAB, for Discriminating Sepsis from SIRS in the ICU. Am J Resp Crit Care Med. 2018;198:903-913. doi: 10.1164/rccm.201712-2472OC.
