# Supplementary material for: The effect of uncertainty in patient classification on diagnostic performance estimations
Source: PLoS One. 2019 May 22;14(5):e0217146. doi: 10.1371/journal.pone.0217146 (PMC6530857; doi:10.1371/journal.pone.0217146)
Supplement: S3 Supporting Information — Using the dataset of Miller et al. [25] and the method described in S2 Supporting information, each patient receives a probabilistic assessment of his or her systemic infection status. Samples are then selected, on the basis of the observed uncertainty distribution. This selection process reflects the expectation that patients with more certainty in classification are less likely to be misclassified. The selected samples are then relabeled with the opposite status indicator. (PDF) [file pone.0217146.s003.pdf]

S3 Supporting Information: Weighting for introduced misclassification events

Using the dataset of Miller et al. [1] and the method described in S2 supporting information, each patient received a probabilistic assessment of his or her systemic infection status. Suppose a patient was assessed with an 87.5% probability of systemic infection. If the trial were run again, assuming all of the expert panelists were equally competent, then for any given clinician providing a diagnosis, this particular patient would be expected to have a 87.5% chance of being diagnosed as 'systemic infection present', and a 12.5% chance of being diagnosed as 'systemic infection absent'.

To reflect a given misclassification rate, for example a 10% false negative (FN) rate, 10% of the known positive samples must be selected and re-labeled as negative. Selection of specific samples to re-label is based on the observed uncertainty distribution. For example, patients with a 75% confident diagnosis of systemic infection have a residual diagnostic uncertainty of 25%, while patients with a 50% confident diagnosis of systemic infection have a residual diagnostic uncertainty of 50%. When randomly selecting patients for misclassification as false positives according to the algorithm and online application described in this paper, those having a residual diagnostic uncertainty of 50% have 2x the likelihood of being selected, compared to those having a residual diagnostic uncertainty of 25%. This weighting scheme reflects the expectation that patients with more certainty in classification are less likely to be misclassified.

References

1. Miller III RR, Lopansri BK, Burke JP, Levy M, Opal S, Rothman RE, et al. Validation of a Host Response Assay, Septicyte™ LAB, for Discriminating Sepsis from SIRS in the ICU. Am J Resp Crit Care Med. 2018;198:903-913. doi: 10.1164/rccm.201712-2472OC.
