# Supplementary material for: The effect of uncertainty in patient classification on diagnostic performance estimations
Source: PLoS One. 2019 May 22;14(5):e0217146. doi: 10.1371/journal.pone.0217146 (PMC6530857; doi:10.1371/journal.pone.0217146)
Supplement: S4 Supporting Information — The overall expected total misclassification rate is the FP rate applied to the negative patients plus the FN rate applied to the positive patients. (PDF) [file pone.0217146.s004.pdf]

# S4 Supporting information: Calculating misclassification rates, based on patient confidence values

We refer to Miller et al. [1] for a description of a new diagnostic test for sepsis. The estimated probability of sepsis for each patient in a cohort can be calculated as described in Supporting Information S2. If a patient is assigned an overall probability of sepsis of 90% then he or she can be considered septic with a 10% probability of being misclassified as systemic inflammatory response syndrome, i.e. SIRS (false negative rate = 0.10). Similarly, a patient with a 33% probability of sepsis can be considered SIRS with a 33% probability of being misclassified as septic (false positive rate = 0.33). Averaging the false negative (FN) rate over all patients gives the overall expected FN rate, and similarly averaging the false positive (FP) rate over all patients gives the overall expected FP rate. The overall expected total misclassification rate is the FP rate applied to the negative patients plus the FN rate applied to the positive patients. For example, if the sum of FP rates over all patients is 5.33% and there are 100 patients classified as negative, and the sum of FN rates over all patients is 6.23% and there are 200 patients classified as positive, then the overall expected total misclassification rate is (5.33%*100+6.23%*200)/(100+200)=5.93%.

## References

1. Miller III RR, Lopansri BK, Burke JP, Levy M, Opal S, Rothman RE, et al. Validation of a Host Response Assay, Septicyte™ LAB, for Discriminating Sepsis from SIRS in the ICU. Am J Resp Crit Care Med. 2018;198:903-913. doi: 10.1164/rccm.201712-2472OC.
