# Supplementary material for: The effect of uncertainty in patient classification on diagnostic performance estimations
Source: PLoS One. 2019 May 22;14(5):e0217146. doi: 10.1371/journal.pone.0217146 (PMC6530857; doi:10.1371/journal.pone.0217146)
Supplement: S5 Supporting Information — A set of simulation runs was conducted to further explore the effect, on apparent performance of an index test, of 5% noise injected into the comparator. (PDF) [file pone.0217146.s005.pdf]

S5 Supporting information: Decrease in apparent performance of index test, with 5% noise injected into comparator

A set of simulation runs was conducted to further explore the effect, on apparent performance of an index test, of 5% noise injected into the comparator. The particular question addressed here is the dependence of p-value (significance estimator) on the number of simulated samples (trial size). It is expected that the confidence interval (CI) around each performance parameter's point estimate will shrink as the number of simulated samples is increased. Therefore, it is reasonable to hypothesize that the p-value for a difference between performance point estimates (for 0% versus 5% misclassification in the comparator) will also decrease, with increasing numbers of simulated samples.

Statistical tests for significance (p-value) were as follows: for AUC, DeLong's test in the R package pROC v1.14.0; and for all other parameters, a 2-sample test for equality of proportions with continuity correction, using function prop.test in the R package stats v3.5.3.

Results (p-value for difference between performance point estimates) are presented in Table S5.1 and Figure S5.1. With 2000 simulated samples, all p-values have decreased to <0.05.

Table S5.1 Dependence of p-value on number of simulated samples.

	n=200 (seed 1)	n=200 (seed 2)	n=400	n=600	n=800	n=1000	n=2000
**pval_AUC**	0.0433	0.02873	0.002044	0.004164	0.0009948	0.0003924	<0.0001
**pval_Sens**	0.3142	0.6096	0.1584	0.1344	0.0778	0.09803	0.01531
**pval_Spec**	0.3932	0.497	0.2309	0.2982	0.1975	0.138	0.03017
**pval_PPV**	0.363	0.4593	0.2334	0.3378	0.2234	0.1362	0.02881
**pval_NPV**	0.3286	0.6433	0.1568	0.117	0.06811	0.09918	0.01594

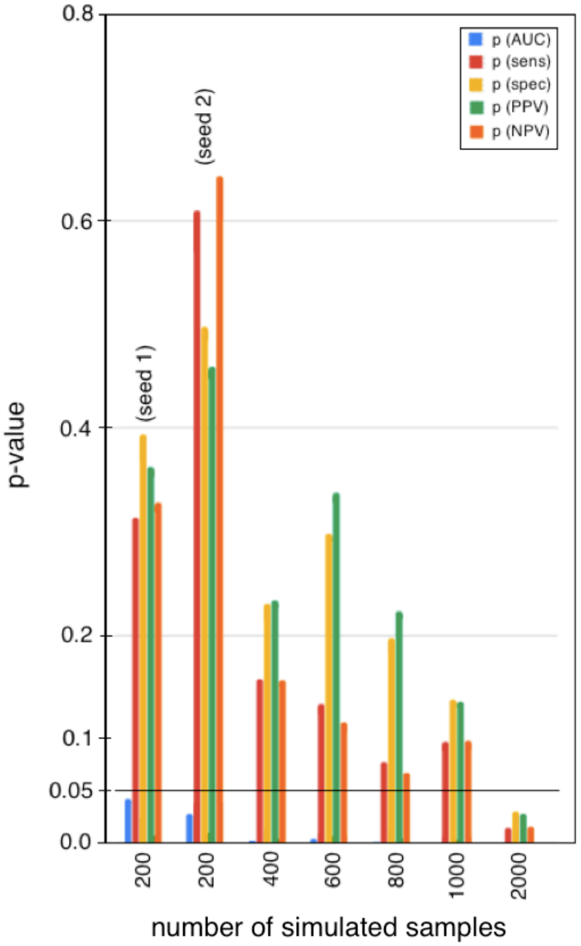

Fig S5.1 Dependence of p-value on number of simulated samples.
