# Supplementary material for: The effect of uncertainty in patient classification on diagnostic performance estimations
Source: PLoS One. 2019 May 22;14(5):e0217146. doi: 10.1371/journal.pone.0217146 (PMC6530857; doi:10.1371/journal.pone.0217146)
Supplement: S6 Supporting Information — The examples show degradation of apparent performance of a diagnostic test as a function of noise in the comparator, when FP and FN rates are not equal. (PDF) [file pone.0217146.s006.pdf]

S6 Supporting information: Unequal FP and FN rates

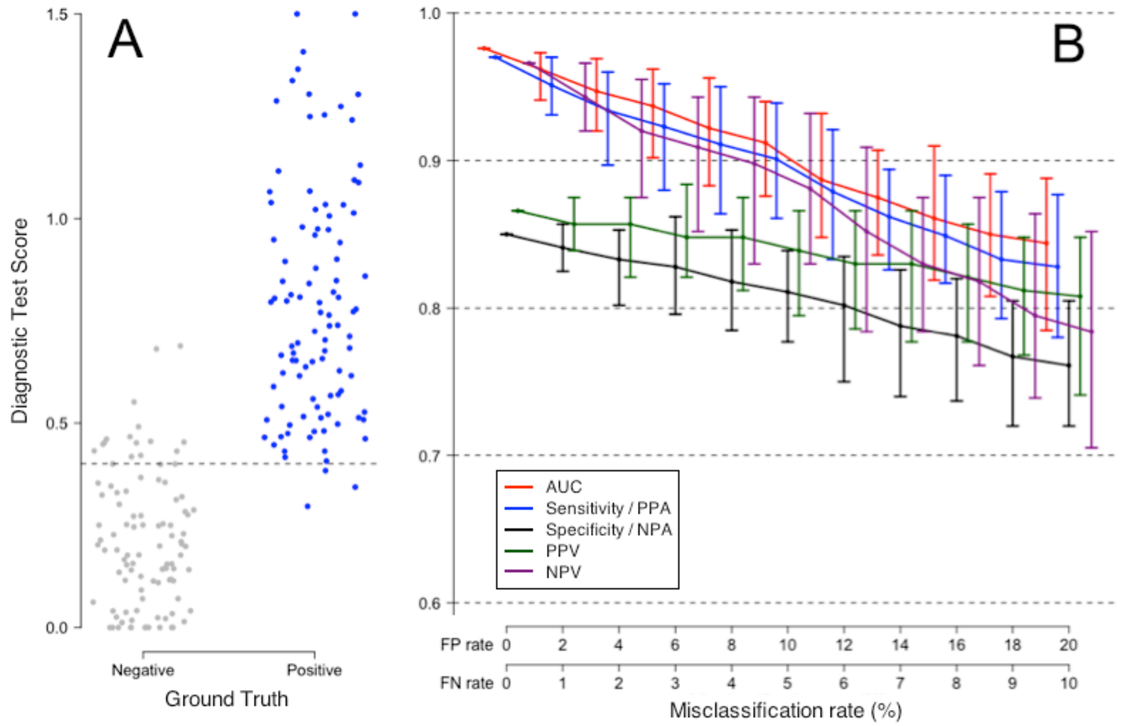

Fig S6.1 Degradation of apparent performance of a diagnostic test as a function of noise in the comparator, when FP rate > FN rate. (A) This panel reproduced from Figure 4 in the main text. Diagnostic test score (y-axis) compared to the Ground Truth, showing the true test performance. Grey points: 100 True Negatives at the level of Ground Truth. Blue Points: 100 True Positives at the level of Ground Truth. A slight overlap between Ground Truth negative and Ground Truth positive distributions is assumed, leading to AUC 0.98 with the Ground Truth as reference. (B) Apparent performance of diagnostic test, as a function of the comparator's misclassification rate. In the scenario described by panel B, FP rate = 2x FN rate. In other words, Ground Truth negative patients are misclassified as positive at twice the rate that Ground Truth positive patients are misclassified as negative. The terms Sensitivity and Specificity are appropriate when there is no misclassification in

the comparator (FP rate = FN rate = 0%). The terms Positive Percent Agreement (PPA) and Negative Percent Agreement (NPA) should be used in place of Sensitivity and Specificity, respectively, when the comparator is known to contain uncertainty.

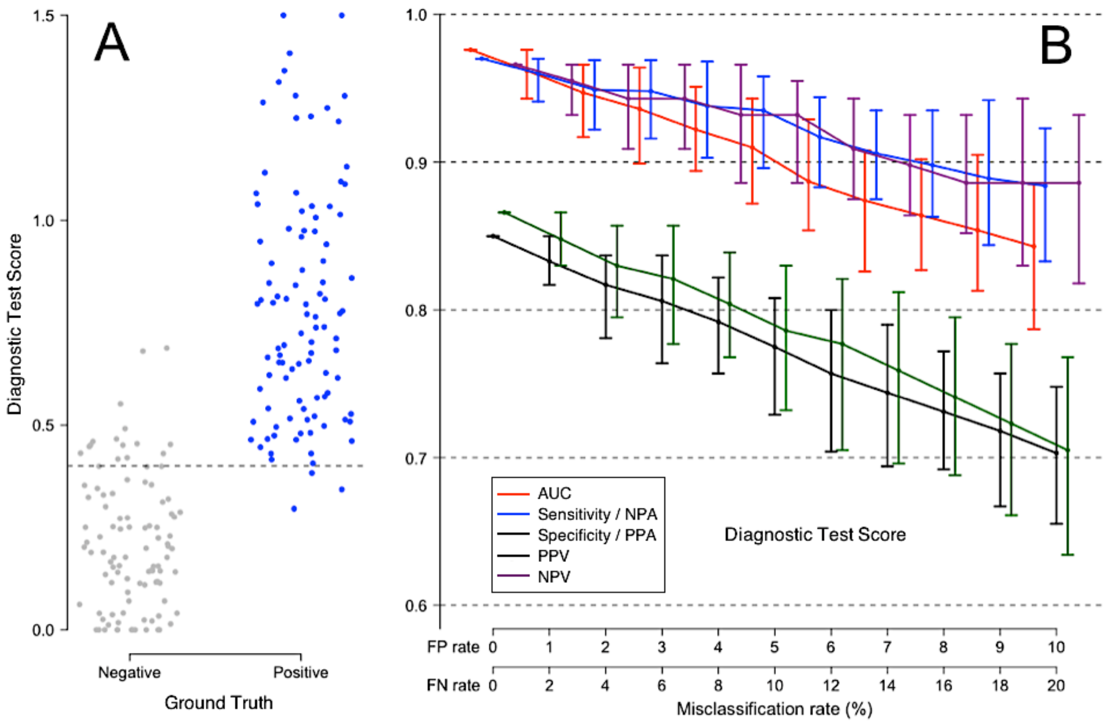

Fig S6.2 Degradation of apparent performance of a diagnostic test as a function of error in the comparator, when FN rate > FP rate. (A) This panel reproduced from Figure 4 in the main text. Diagnostic test score (y-axis) compared to the Ground Truth, showing the true test performance. Grey points: 100 True Negatives at the level of Ground Truth. Blue Points: 100 True Positives at the level of Ground Truth. A slight overlap between Ground Truth negative and Ground Truth positive distributions is assumed, leading to AUC 0.98 with the Ground Truth as reference. (B) Apparent performance of the diagnostic test, as a

function of the comparator's misclassification rate. In the scenario described by panel B, FN rate = 2x FP rate. In other words, Ground Truth positive patients are misclassified as negative at twice the rate that Ground Truth negative patients are misclassified as positive. The terms Sensitivity and Specificity are appropriate when there is no misclassification in the comparator (FP rate = FN rate = 0%). The terms Positive Percent Agreement (PPA) and Negative Percent Agreement (NPA) should be used in place of Sensitivity and Specificity, respectively, when the comparator is known to contain uncertainty.
