# Supplementary material for: The effect of uncertainty in patient classification on diagnostic performance estimations
Source: PLoS One. 2019 May 22;14(5):e0217146. doi: 10.1371/journal.pone.0217146 (PMC6530857; doi:10.1371/journal.pone.0217146)
Supplement: S7 Supporting Information — The analysis shows that the goal of achieving a very high diagnostic test performance, such as a 99% sensitivity, can be rendered nearly unreachable, if the comparator diagnosis contains even small amounts of uncertainty. (PDF) [file pone.0217146.s007.pdf]

S7 Supporting information: Very high performance tests

The following analysis shows that the goal of achieving a very high diagnostic test performance, such as a 99% sensitivity, can be rendered nearly unreachable, if the comparator diagnosis contains even small amounts of uncertainty.

Step 1: Consider a diagnostic test which perfectly discriminates negative and positive subjects, under the assumption that the comparator is noise-free.

Generate simulation data:

1000 negative subjects (mean score 0.20, standard deviation 0.10)

1000 positive subjects (mean score 1.30, standard deviation 0.20)

Use a binary cut-off threshold of 0.6.

This scenario is shown below in Fig S7.1.

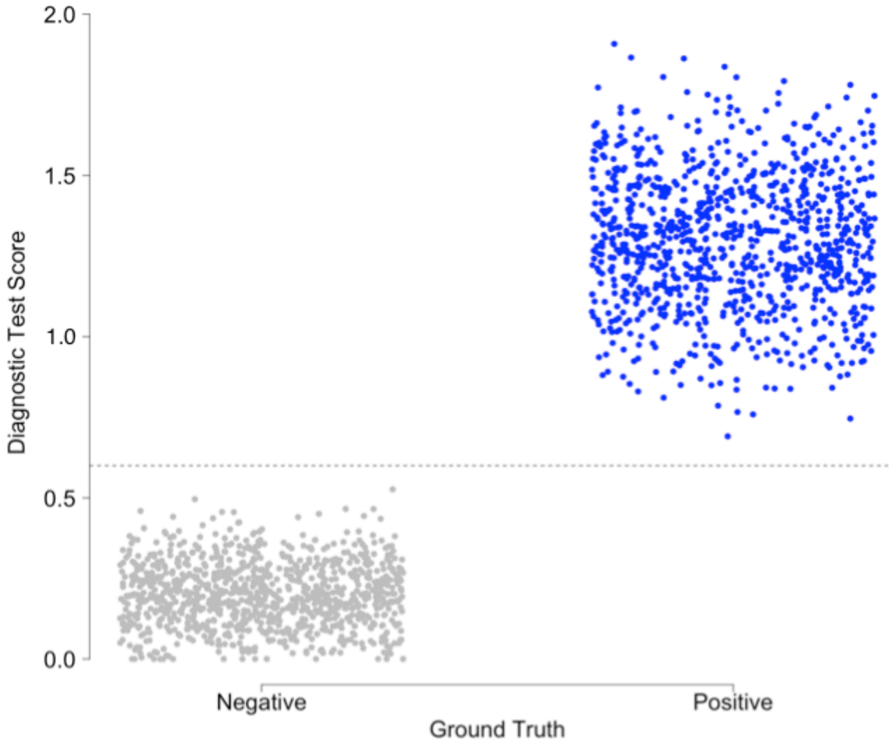

Fig S7.1 Behavior of diagnostic test having perfect separation of negative and positive subjects, with no comparator error. Negative subjects: mean 0.20, SD 0.10. Positive subjects: mean 1.30, SD 0.20.

Step 2: Into the above distribution, randomly introduce a 2.5% false positive rate (FPR) and a 2.5% false negative rate (FNR) in the comparator (a combined 5% misclassification rate).

After 1000 iterations, the following distributions of performance are generated for the diagnostic test in question, as shown in Fig S7.2.

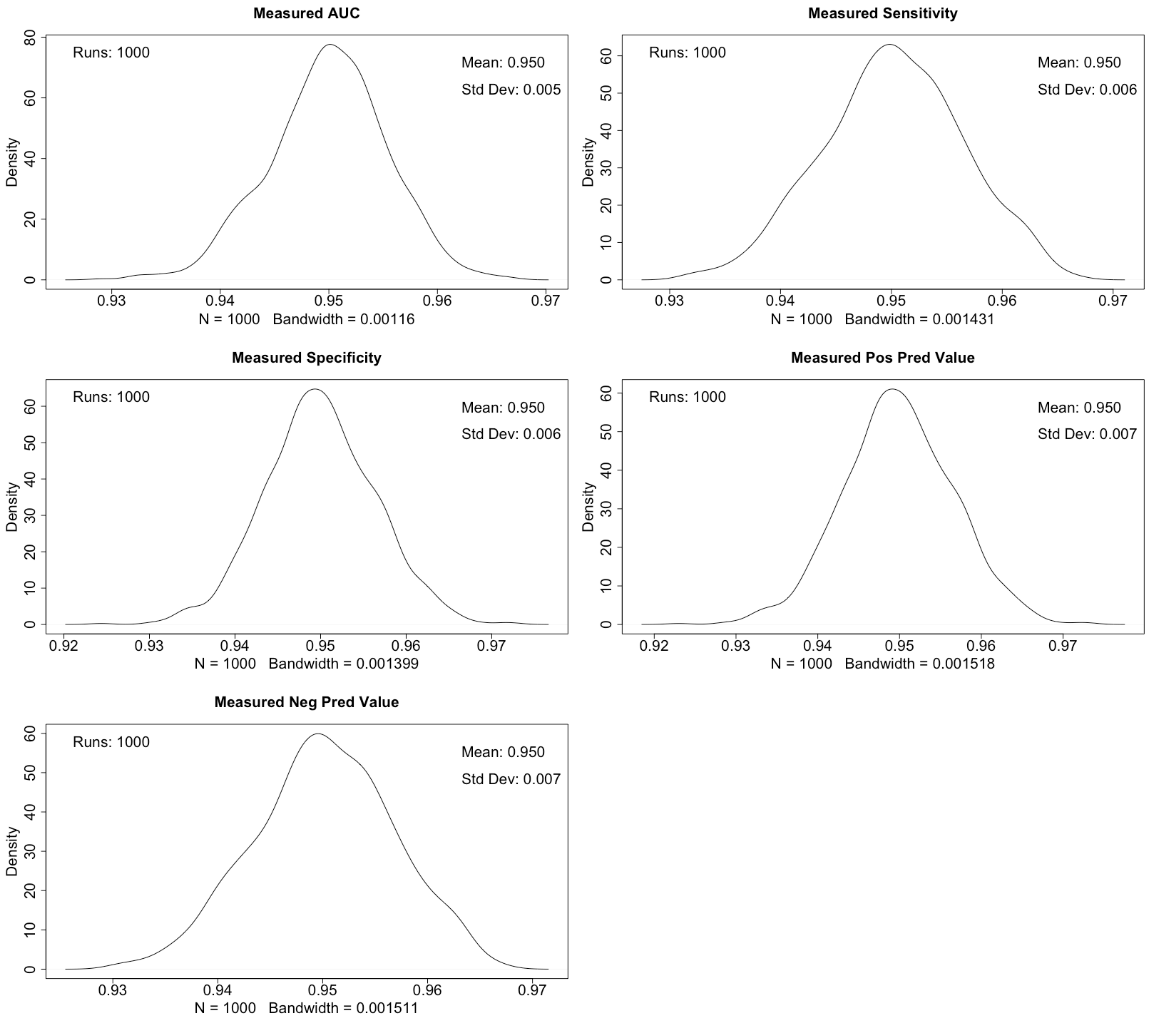

Fig S7.2 Simulated performance results for a perfect diagnostic test, assuming a 5% misclassification rate by the comparator.

From above, we expect an average NPV of 0.950 with a standard deviation of 0.007. If we require a 99% NPV for this perfect test, then (assuming a 5% error rate in the comparator) we may use the normal probability density function to estimate the probability of this occurring.

Step 3: Calculate the probability of measuring a 99% or greater NPV for the perfect test, under the assumption of 5% comparator noise:

(0.99-0.950)/0.007 = 5.572 = number of standard deviations above the mean that 99% cutoff for NPV lies.

The corresponding probability of occurrence is given by the normal probability density function:

$$p(x) = \frac{1}{\sigma\sqrt{2\pi}}\, e^{-\left(\frac{(x-\mu)^2}{2\sigma^2}\right)}$$

This can be calculated using the following online app:

https://www.fourmilab.ch/rpkp/experiments/analysis/zCalc.html

Given x = 5.572, the chance probability p(x) is: 1.26×10^{-8}.
